# Emergency Presentations With Nonspecific Complaints—the Burden of Morbidity and the Spectrum of Underlying Disease: Nonspecific Complaints and Underlying Disease

**DOI:** 10.1097/MD.0000000000000840

**Published:** 2015-07-02

**Authors:** Julia Karakoumis, Christian H. Nickel, Mark Kirsch, Martin Rohacek, Nicolas Geigy, Beat Müller, Selina Ackermann, Roland Bingisser

**Affiliations:** From the Emergency Department, University Hospital, Basel (JK, CHN, MK, MR, SA, RB); Emergency Department, Cantonal Hospital, Liestal (NG); and Emergency Department, Cantonal Hospital, Aarau, Switzerland (BM).

## Abstract

The prevalence of diagnoses, morbidity, and mortality of patients with nonspecific complaints (NSC) presenting to the emergency department (ED) is unknown.

To determine the prevalence of diagnoses, acute morbidity, and mortality of patients with NSC.

Prospective observational study with a 30-day follow-up. Patients presenting to 2 EDs were enrolled by a study team and diagnosed according to the World Health Organization ICD-10 System.

Of 217,699 presentations to the ED from May 2007 through to February 2011, a total of 1300 patients were enrolled. After exclusion of 90 patients who fulfilled exclusion criteria, 1210 patients were analyzed. No patient was lost to follow-up. In patients with NSC, the underlying diseases were spread throughout 18 chapters of the ICD-10. A total of 58.7% of the patients were diagnosed with acute morbidity. Thirty-day mortality was 6.4% overall. Patients with acute morbidity and suffering from heart failure and pneumonia had mortalities >15%; patients lacking acute morbidity, but suffering from functional impairment or depression/anxiety had mortalities of 0%. Although the history did not allow any prediction, age and sex were predictive of morbidity and mortality.

The differential diagnoses in patients presenting with NSC is broad. Acute morbidity and mortality were high in the presented cohort, the predictors of morbidity and mortality being age and sex rather than the nature of the complaints. Urgently needed management strategies could be based on these results.

ClinicalTrials.gov (#NCT00920491).

## Abstract 2

Supplemental Digital Content is available in the text

## INTRODUCTION

Up to 20% of older patients present with nonspecific complaints (NSC), such as generalized weakness, functional impairment, or feeling exhausted.^1^ These are poorly defined symptoms with little discriminative power. Thus, it is challenging to diagnose these patients. The diagnostic work-up tends to be time-consuming and sometimes inefficient, as an initial working diagnosis is difficult to draft,^2–3^ and the possibility of initially missed acute morbidity is high.^2,4–8^ Because of physiological changes in the elderly, acute diseases often present nonspecifically,^2,5–8^ and the prevalence of an acute medical disorder in patients with NSC ranges from 51%^2^ to 59%.^4^ As older patients are the fastest growing population in acute care, NSC will gain importance. Previous investigations have discussed the differential diagnoses of weakness, functional impairment, and dizziness,^5–8^ but there is no prospective observational trial of patients with NSC presenting to emergency departments (EDs).

Thus, we performed a prospective observational study to determine the prevalence of underlying diseases in patients presenting with NSC, and to measure their acute morbidity and mortality rates.

## METHODS

### Study Design

We conducted a prospective observational study with a 30-day follow-up. The study protocol was approved by the local ethics committee “Ethikkommission beider Basel” and performed in compliance with the Helsinki Declaration. All participants gave their written informed consent.

Diagnoses were classified according to the World Health Organization ICD-10 System (International Statistical Classification of Diseases and Related Health Problems, 10th Revision).

### Study Setting and Population

The study took place in the EDs of Basel University Hospital, Switzerland, which is a tertiary care hospital, and in the ED of the Hospital of Liestal, Switzerland, a secondary care hospital. Nontrauma patients older than 18 years and presenting with NSC between May 24th 2007 and February 2nd 2011 were screened for inclusion. The Emergency Severity Index (ESI)^9^ was used to exclude all patients in need of a life-saving intervention (ESI 1), and patients needing a focused assessment only (ESI 4 or 5).

### Inclusion and Exclusion Criteria

Patients were eligible if they presented with NSC—defined as all complaints outside the set of specific complaints for which evidence-based management protocols for emergency physicians exist.^4^ Typical examples of NSC were generalized weakness, feeling exhausted, fatigue, recent falls, or dizziness.

Patients were included by the study team after history taking and after focused clinical examination, but before laboratory results were available. Patients with the following conditions were not included:

1. specific complaints, such as chest pain or dyspnoea
2. clinical presentations suggestive of a working diagnosis to be managed by evidence-based protocols (eg presenting with a main complaint of weakness, but showing an obvious anemic pallor)
3. ESI score of 1, 4, or 5
4. vital signs significantly out of range (systolic blood pressure <90 mm Hg, heart rate >120 beats/min, tympanic body temperature >38.4°C or <35.6°C, respiratory rate >30 breaths/min, oxygen saturation (SpO_2_) <92%)
5. recent external laboratory results, or referral from other hospitals
6. specific electrocardiogram changes on admission (ST-segment elevation)
7. moribund patients with terminal conditions (cachexia in end-stage cancer)
8. patients who did not sign an informed consent form

### Measurements

Demographic baseline data (date of birth, sex), ESI-level,^9^ mode of admission (self-referral, by family doctor, by proxy, by ambulance/EMS, and others), current complaints (using predefined structured data sheets), vital signs (heart rate, temperature, blood pressure, Glasgow Coma Scale, oxygen saturation [SpO_2_], and respiratory rate), physical examination, living situation (at home and independent, at home with the help from family or neighbors, at home with professional help, nursing home), comorbidities,^10^ all concomitant drugs, and electrocardiogram-findings were obtained.

### Outcomes

Outcome measures were death, cause of death by autopsy results, and acute mortality within a 30-day follow-up. According to our framework,^4^ acute morbidity was defined as a serious condition, that is, any condition requiring early intervention (eg the use of antibiotics) to avoid deterioration of health status, possibly leading to adverse health outcomes such as disability, or death.

Outcome ascertainment was performed after a 30-day follow-up, using hospital discharge letters and questionnaires from family physicians. Data were independently analyzed by outcome assessors, 2 physicians certified in internal medicine and emergency medicine. Final underlying diagnoses according to ICD-10 and the outcomes were determined. In case of disagreement, an expert panel was consulted.

### Diagnoses According to the ICD-10 Classification

ICD was choosen as classification system, as it has been used in World Health Organization member countries since 1994^11^ and uses stringent rules. After reviewing and analyzing all patient charts and physician reports, the outcome assessors made a clinical diagnosis and attributed ICD-10 codes to the primary and secondary underlying diagnosis according to ICD-10 rules.

Rare diagnoses (<3 cases) were named “other” within their respective chapters.

### Defining Clinical Diagnostic Groups

For clinical purposes, an amalgamation to clinical diagnostic groups was performed, aggregating ICD-10 subgroups across chapters, as defined below:

All types of dementia (F00-F03, I67, G30.1) coded depending on etiology (circulatory system or nervous system) were taken together as “dementia.”

All strokes coded as “circulatory” (I61-I66, I69), “external cause” (S06), or “nervous system” (G45) were taken together as “cerebral hemorrhage or cerebral ischemic disease.”

Infections within chapter I ([A04, A08-A09], bacteremia [A40-A41], unspecified infections [B99]), and rare infections of various organs (nervous system, ear, circulatory system, skin, and muscular-skeletal system) were taken together as “other infections.”

Separate entities included pneumonia (J13-J18), bronchitis/COPD (J20-J44), peritonitis (K63-K65), and urinary tract infection (N30, N39).

In cases where a final ICD-diagnosis could not be established, the presenting clinical syndrome was coded. Functional impairment, for example, was attributed to R53/R54.

### Statistical Analysis

Statistical analysis was performed. To analyze baseline characteristics, the nonparametric Mann-Whitney test was used to compare the median age of the male and female cohorts (*P* values of ≤0.05 being statistically significant). Median and interquartile ranges of nonnormally distributed categorical and metric variables of the study population (ie, age, Charlson index, and number of concomitant drugs) were assessed. All normally distributed dichotomous and categorical variables of the whole cohort (ie, number of male/female patients, living situation, and ESI-score) are expressed as counts (percentages).

The prevalence of diagnoses according to the ICD-10 System was analyzed. In an additional step, the 12 most frequent clinical diagnostic groups were identified. The prevalence rates within the cohort, according to sex and age (young [<65-year olds], young old [65–74-year olds], middle old [75–84-year olds], and oldest old patients [≥85-year olds]) with corresponding 95% confidence intervals were analyzed. Significant differences between these groups in the prevalence of the 12 most common clinical diagnostic groups were identified by the 2-sided chi-square (^2^) test. All tests were performed using significance levels of α = 0.05 (^∗^), α = 0.01 (^∗∗^), and α = 0.001 (^∗∗∗^), respectively.

Furthermore, the prevalence of acute morbidity, and death within 30-day follow-up was assessed in relation to the 12 most frequent clinical diagnostic groups.

The prevalence of acute morbidity and death within follow-up were compared within the cohort having a frequent (1 of the 12 most common clinical diagnostic groups), versus an uncommon diagnosis (outside the set of the 12 most common clinical diagnostic groups) by the 2-sided chi^2^ test. Statistical analyses were performed using SPSS 20.0 (SPSS, Chicago, IL). Excel 2010 for Windows was used for prevalence bar plots with 95% confidence intervals.

## RESULTS

Of 217,699 presentations to the ED from May 24th 2007 through to February 2nd 2011, a total of 1300 patients were enrolled. After exclusion of 90 patients who fulfilled exclusion criteria, 1210 patients were analyzed. Table 1 shows the patient's baseline characteristics. A total of 468 (38.7%) were male and 742 (61.3%) were female (gender ratio 1:1.6). Median age was 81 years. Male patients were significantly younger than female patients (79 vs 83 years; *P* < 0.001). A total of 1061 (87.1%) patients were triaged as ESI 3, and 48 (4%) patients were triaged as ESI 2. The discharge rate from the ED was 10%. 2.5% of the discharged patients died within 30 days.

**TABLE 1:**
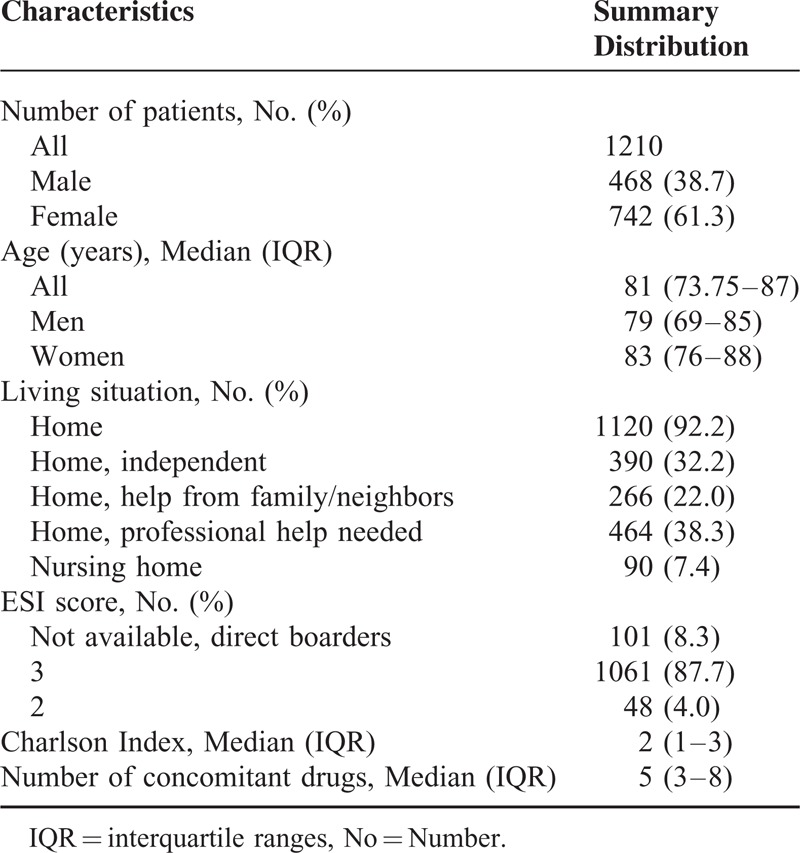
Baseline Patients Characteristics

## DISTRIBUTION OF DIAGNOSES ACCORDING TO THE ICD-10 CLASSIFICATION

Underlying diagnoses of all patients were classified across 18 of 22 ICD-10 Chapters (see Supplementary Appendix, Table S1, http://links.lww.com/MD/A325). Prevalent codes were in chapter IX (Diseases of the circulatory system, I00-I99; N = 191), chapter V (Mental and behavioral disorders, F00-F99; N = 182), and chapter XIV (Diseases of the genitourinary system, N00-N99; N = 171).

Codes of 91 patients of the cohort (7.5%) were assigned to 1 of the 13 “other”-subgroups.

## PREVALENCE OF DIAGNOSES ACCORDING TO CLINICAL DIAGNOSTIC GROUPS

**FIGURE 1:**
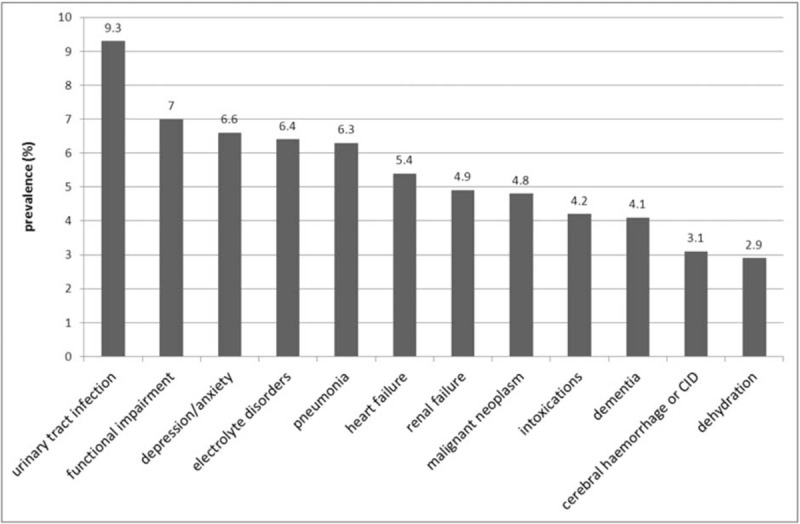
Prevalence (in percent) of the most frequent clinical diagnoses or clinical diagnostic groups in the cohort (N = 1210) in descending order. Patients with nonspecific complaints (NSC) most often suffered from urinary tract infection, functional impairment, or depression/anxiety. CID = cerebral ischemic disease.

We amalgamated 50 clinical diagnostic groups (Table S1, http://links.lww.com/MD/A325). The 12 most prevalent (Figure 1) accounted for 65% (N = 786) of all primary diagnoses. The most frequent clinical diagnostic group was urinary tract infection (9.3%), followed by functional impairment (7%), and depression/anxiety (6.6%).

## PREVALENCE OF DIAGNOSES ACCORDING TO GENDER

**FIGURE 2:**
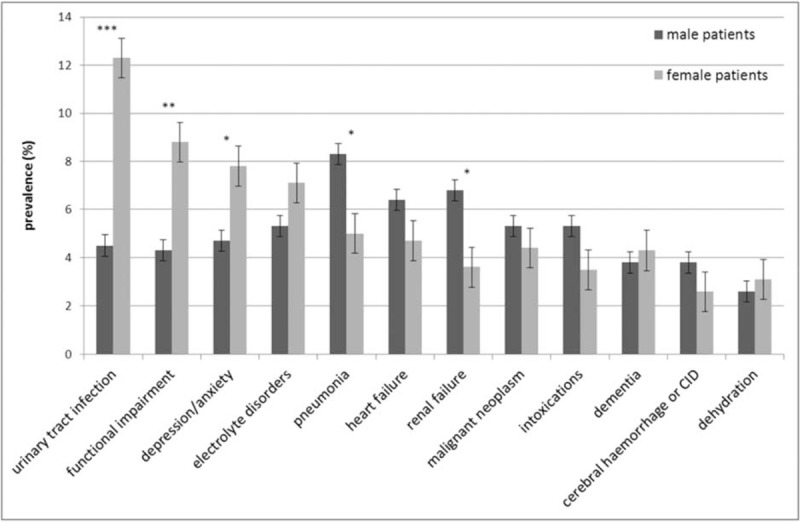
Comparison of prevalence rates (in percent) of the most frequent clinical diagnoses or clinical diagnostic groups in male (N = 468) and female (N = 742) patients of our cohort in descending order. Male patients suffered considerably more often from pneumonia (*P* = 0.02), and renal failure (*P* = 0.01) than the female cohort. Female patients suffered significantly more often from urinary tract infection (*P* < 0.001), functional impairment (*P* = 0.003), and depression/anxiety (*P* = 0.03) than the male cohort. Significant differences are highlighted by asterisks (^∗^ = *P* ≤ 0.05; ^∗∗^ = *P* < 0.01; ^∗∗∗^ = *P* < 0.001). CID = cerebral ischemic disease.

Significant differences between men and women were observed (Figure 2): urinary tract infection (*P* < 0.001), functional impairment (*P* = 0.003), depression/anxiety (*P* = 0.03), pneumonia (*P* = 0.02), and renal failure (*P* = 0.01). The prevalence of the other clinical diagnoses or clinical diagnostic groups was not significantly dependent on gender.

The most frequent clinical diagnosis in males was pneumonia (N = 39 of 468; 8.3%), followed by renal failure (N = 32, 6.8%), as opposed to urinary tract infection (N = 91 of 742; 12.3%), functional impairment (N = 65; 8.8%), and depression/anxiety (N = 58; 7.8%) in females.

## PREVALENCE OF DIAGNOSES ACCORDING TO AGE

**FIGURE 3:**
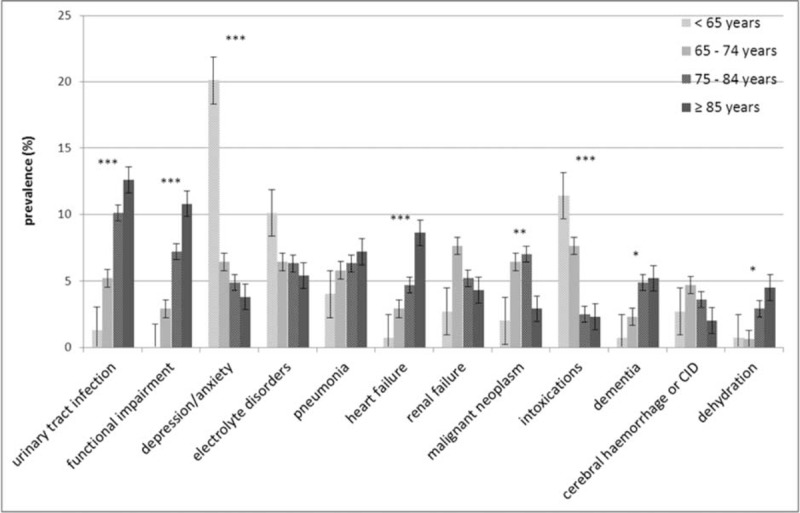
Comparison of prevalence rates (in percent) of the most frequent clinical diagnoses or clinical diagnostic groups in young (<65-year old; N = 149), young old (65–74-year old; N = 172), middle old (75–84-year old; N = 445), and oldest old (≥85-year old; N = 444) patients of our cohort in descending order. The prevalence of urinary tract infection (*P* < 0.001), functional impairment (*P* < 0.001), heart failure (*P* < 0.001), dementia (*P* = 0.05), and dehydration (*P* = 0.02) was considerably higher in older patients than in younger patients. The prevalence of depression/anxiety (*P* < 0.001) and intoxications (*P* < 0.001) was significantly higher in younger patients than in older patients. The prevalence of malignant neoplasm (*P* = 0.009) was significantly higher in young old and middle old patients than in young and oldest old patients. Significant differences are highlighted by asterisks (^∗^ = *P* ≤ 0.05; ^∗∗^ = *P* < 0.01; ^∗∗∗^ = *P* < 0.001). CID = cerebral ischemic disease.

Certain diagnostic groups showed age-dependent prevalence rates (Figure 3): urinary tract infection (*P* < 0.001), functional impairment (*P* < 0.001), depression/anxiety (*P* < 0.001), heart failure (*P* < 0.001), malignant neoplasm (*P* = 0.009), intoxications (*P* < 0.001), dementia (*P* = 0.05), and dehydration (*P* = 0.02).

Prevalent conditions among >85-year-old patients were urinary tract infection (N = 56 of 444; 12.6%), functional impairment (N = 48; 10.8%), and heart failure (N = 38; 8.6%). Prevalent conditions among 75 to 84-year-old patients were urinary tract infection (N = 45 of 445; 10.1%), functional impairment (N = 32; 7.2%), and malignant neoplasm (N = 31; 7%). Prevalent conditions among 65 to 74-year-old patients were renal failure (N = 13 of 172; 7.6%), intoxications (N = 13; 7.6%), and malignant neoplasm (N = 11; 6.4%). Prevalent conditions among <65-year-old patients were depression/anxiety (N = 30 of 149; 20.1%), intoxications (N = 17; 11.4%), and electrolyte disorders (N = 15; 10.1%).

## OUTCOMES ACCORDING TO CLINICAL DIAGNOSTIC GROUPS

### Acute Morbidity

Acute morbidity occurred in 710 of 1210 patients (58.7%); 302 (64.5%) in males and 408 (55%) in females (*P* = 0.001). Seventy five to 84-year-old patients had the highest prevalence of acute morbidity (N = 271 of 445; 60.9%), as opposed to patients younger than 65 years with the lowest prevalence of acute morbidity (N = 74 of 149; 49.7%; *P* = 0.02). Within the 12 most prevalent clinical diagnostic groups, 58.1% (N = 457 of 786) suffered from acute morbidity, most commonly on account of the following clinical diagnostic groups (Table 2): heart failure (100%), pneumonia (98.7%), renal failure (98.3%), electrolyte disorders (97.4%), cerebral haemorrhage or cerebral ischemic disease (86.5%), dehydration (85.7%), malignant neoplasm (77.6%), and urinary tract infection (53.6%).

**TABLE 2:**
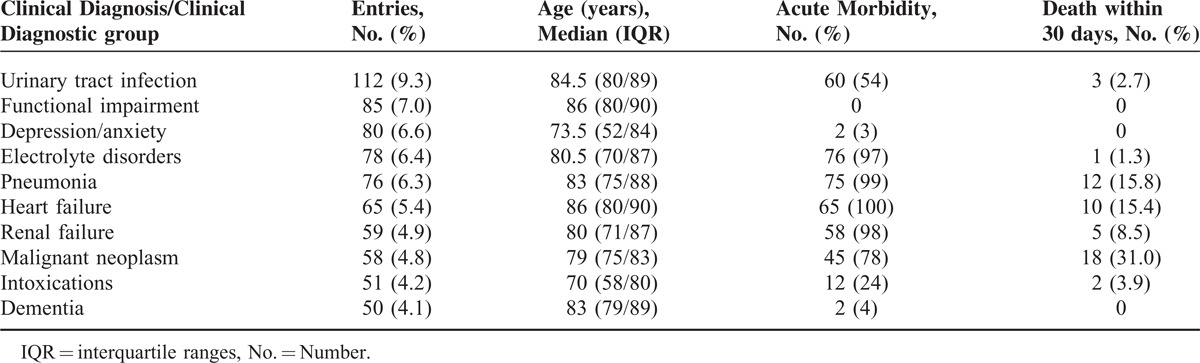
Rate of Morbidity and Death Within 30 Days in Relation to the Most Frequent Clinical Diagnoses

The prevalence of serious conditions was comparable between the 12 most common clinical diagnostic groups (N = 457 of 786; 58.1%), and the remainder (N = 253 of 424; 59.7%; *P* = 0.61).

### Mortality (30 Days)

Seventy-seven patients (6.4%) died within follow-up. Male patients (N = 43; 9.2%) had a higher mortality than female patients (N = 34; 4.6%; *P* = .001). Seventy five to84-year-old patients had the highest 30-day mortality (N = 35; 7.9%), as compared with patients younger than 65 years with the lowest mortality (N = 4; 2.7%; *P* = .03). High mortalities were observed in malignant neoplasm (31%), pneumonia (15.8%), and heart failure (15.4%).

Mortality did not significantly differ between the 12 most common clinical diagnostic groups (N = 52 of 786; 6.6 %) as compared to the remainder (N = 25 of 424; 5.9%; *P* = 0.63).

## DISCUSSION

The main findings were the broad diagnostic spectrum of underlying disease, the high morbidity and mortality, and the effects of age and gender on the distribution in the diagnostic spectrum.

The first new finding was that the spectrum of underlying diagnoses was widely spread throughout 18 of 22 ICD-10 chapters, covering the areas of internal medicine, neurology, geriatrics, and psychiatry. Better knowledge about the prevalence of underlying disease is of practical use, as it is the basis for the development of comprehensive diagnostic protocols. Such standardized work-up needs to be economically and medically efficient, prohibiting the often observed “wait-and-see” and “do-all-tests” strategies.

Secondly, in spite of their nonspecific presentation, 59% of all patients suffered from acute morbidity, in need of early interventions to prevent health status deterioration, and 6.4% of the patients died within 30 days—the highest mortalities being due to malignant neoplasm, pneumonia, and heart failure. These findings may be used for the development of risk stratification tools, as it is known that a work-up in elderly patients with NSC can become a lengthy and cumbersome affair. Therefore, early risk stratification may aid decisions, for example, disposition planning. If emergency physicians are aware of a low short-term mortality, there is a higher likelihood of discharge. If predicted short-term mortality exceeds for example 10%, disposition to acute care facilities is warranted.

Thirdly, male and older patients had a higher prevalence of acute morbidity (eg, pneumonia or heart failure) and higher mortality, as compared with female and younger patients who suffered most commonly from less severe diseases (eg, functional impairment or depression/anxiety). This knowledge is valuable to all clinicians in acute care, as pretest probabilities may be established for age- and gender-subgroups, aiding in the interpretation of test results.

If the most prevalent clinical diagnostic groups overall are considered, the high prevalence of urinary tract infection and pneumonia was no surprize. The blunting of temperature and white-blood cells, in response to serious infectious diseases in the older population^12–16^ (a reason for nonspecific presentation), has been suggested to be a result of decreases in humoral and cellular immunity, making diagnosis more difficult, because the subdued response seems to be associated with atypical presentation.^7^

On the other hand, mortality from pneumonia was 2.5-fold higher in our cohort than the reported mortality of patients hospitalized for pneumonia in Switzerland (15.8% vs 6.4%).^17^ This is partly explained by the higher age of the cohort, but the diagnostic and therapeutic delay due to the nonspecificity of presenting symptoms may account for another part of the increased mortality, as ED boarding time may be associated with higher inpatient mortality rates.^18^

Another explanation may be that nonspecific presentation tends to be associated with higher mortality, as was shown for myocardial infarction, the reasons remaining unclear.^19^

Similarly to infectious diseases, it has been reported that diseases of the circulatory system often present atypically in the elderly.^5,7,17,20^ Thus, it may not seem surprizing that heart failure was among the 6 most prevalent diseases (with the third highest mortality rate of 15.4%) in our cohort.

Although conditions such as infections (urinary tract infection or pneumonia), heart and renal failure, electrolyte disorders, malignant neoplasm, intoxications, and cerebral haemorrhage or ischemic disease were judged to be serious in a large proportion of patients, and had 30-day mortalities of 1.3% to 31%, other conditions, such as functional impairment, depression/anxiety, and dementia had no short-term mortality.

With a median age of 86 years, “functionally impaired” patients were older than the remainder of the cohort (median age of 81 years). As this is the 2nd most prevalent condition, accounting together with depression/anxiety, and dementia, for 18% of all emergency presentations with NSC, it could be of value to focus the primary assessment on these “benign conditions” in order to make early disposition decisions (eg, to geriatric community hospitals or ambulatory care). Unfortunately, these conditions tend to be diagnosed only after prolonged assessments, as they often need exclusion of the vast majority of serious conditions found in our cohort.

The relationship between NSC and mental and behavioral disorders, such as depression and anxiety, has been suggested in previous studies.^5,21–24^ They are the 2 psychiatric conditions most frequently encountered in primary care,^21^ and are present in up to one third of older patients presenting as an emergency.^25,26^ It seems noteworthy that depression/anxiety is the most prevalent clinical diagnostic group (>20%) in our “young” cohort below 65 years of age, but is relatively uncommon (<5%) in patients over 75 years of age.

Findings comparable with published cohorts are the case mix regarding gender, age, morbidity, and mortality^1,2,4,27^ underscoring the feasibility of the inclusion criteria and the definitions used for NSC in our multicentre study.

Furthermore, previous reports have shown that patients who suffer from, for example, generalized weakness can create diagnostic frustration for the primary care physician,^5^ because the diagnostic value of a symptom diminishes with the number of its potential interpretations. Thus, poorly defined symptoms, for example generalized weakness, have little discriminative power in establishing a medical diagnosis.^28^ If physicians are uncertain about the exact nature of symptoms, they must take multiple competing interpretations of the same set of complaints into account.^29^ Therefore, the possibility for diagnostic and therapeutic delay or even misdiagnosis increases.

## STUDY LIMITATIONS

First, this study reflects the reality of 2 Swiss teaching hospitals, which may limit external validity and generalizability to other countries and health systems.

Second, although the predominance of females in our cohort (gender ratio 1.6:1), who were significantly older than the presenting males (83 vs 79 years), can be partly explained by demographics, mirroring the elderly population of Switzerland (gender ratio 1.4:1),^30^ there are still unexplained gender differences in emergency presentations with NSC, such as the remaining overrepresentation of females. Some studies suggest that women suffer more frequently from NSC than men.^31,32^ Furthermore, our male cohort had significantly higher acute morbidity (64.5% vs 55%), and a significantly higher 30-day mortality (9.2% vs 4.6%). This finding is consistent with previous reports of higher hospitalization rates for acute morbidity in men in Switzerland, for example, pneumonia or heart failure.^17,23,33^ Nevertheless, an inclusion bias cannot be completely excluded, as the hospitalization of elderly women may occur with different triggers, possibly due to their role as caregivers or 3-fold higher chance of being widowed.

Third, the outcome assessment might be limited regarding the determination of underlying diseases, as it was strictly based on patient records; some degree of incorporation bias therefore cannot be excluded. However, the direction of such bias is not obvious, as the interrater reliability of the method was assessed in previous studies.^4,34^ Furthermore, the composition of our patient cohort regarding underlying diagnoses, serious condition, and mortality within follow-up was similar to that in our previous studies.^4,34^ Finally, our framework is not validated. However, the framework used is the only one available, and has been used in several studies due to its feasibility. In the 3 clinical diagnostic groups with high mortality (>15%), the prevalence of serious outcome was also high (77.6% to 100%)—taking the 3 clinical diagnostic groups with no mortality, the prevalence of serious outcome was low (0%–4%).

## CONCLUSIONS

On the basis of inferences from our cohort, the prevalence of underlying disease in emergency presentation of patients with NSC could be determined for the first time. For physicians in acute care, the extremely broad spectrum of differential diagnoses is important to acknowledge, and the high proportion of acute morbidity, as well as the 30-day mortality of 6.4%, is noteworthy. Risk factors for adverse health outcomes (acute morbidity and death) need to be determined in order to be used for early therapeutic intervention, and research on risk stratification tools and management protocols is urgently needed for this vulnerable population.

## Acknowledgements

*Professor R. Bingisser had full access to all of the data in the study and takes responsibility for the integrity of the data and the accuracy of the data analysis. The hypothesis of this study arouse before the beginning of the collection of the data. The study protocol was written before the beginning of the collection of the data. The authors thank Dr Rodney Yeates for reviewing the manuscript regarding correct English writing. R. Yeates received wages for their contributions*.
